# Factors influencing multicultural acceptance of Korean nursing students

**DOI:** 10.1186/s12912-023-01583-4

**Published:** 2023-11-14

**Authors:** Minkyung Gu, Sohyune Sok

**Affiliations:** 1 Department of Nursing, College of Health Science, Daejin University, Pocheon-Si, Gyeonggi-Do Republic of Korea; 2 College of Nursing Science, Kyung Hee University, 26, Kyungheedae-Ro, Dongdaemun-Gu, Seoul, 02447 Republic of Korea

**Keywords:** Multicultural acceptance, Empathy, Attitude, Nursing student

## Abstract

**Background:**

South Korea has already become a multicultural society due to immigration, marriage, and employment of foreigners, and the use of medical institutions by foreigners is increasing. In order to establish a therapeutic rapport relationship with foreigners, it is necessary to study multicultural acceptance. This study aimed to examine and identify the factors influencing the degree of multicultural acceptance of Korean nursing students.

**Methods:**

This study used a cross-sectional descriptive design. Study participants included 147 nursing students at University in Seoul or Gyeonggi-do, South Korea. Data included demographics, multicultural acceptance, cultural empathy, and multicultural attitude. Data were collected from March to June 2021. Data was analyzed using linear regression model in SPSS PC + version 23.0 statistical software program.

**Results:**

The adjusted R-squared representing the explanatory power of the model was 72.5%. The strongest predictor was cultural empathy (β = 0.55, *P* < 0.001), followed by multicultural attitude (β = 0.26, *P* = 0.001), residential area where they lived for the past 5 years (β = 0.19, *P* = 0.001), accessibility to multicultural internet and media (β = 0.17, *P* = 0.003), whether they have foreign friends (β = 0.16, *P* = 0.003), and multicultural education experience (β = 0.02, *P* = 0.002).

**Conclusion:**

This study suggests that to improve the degree of multicultural acceptance of Korean nursing students, their cultural empathy and multicultural attitude should be strengthened. Nurses need to pay attention the influencing factors to improve the multicultural acceptance of Korean nursing students.

## Background

The inflow of foreigners to Korea for various purposes such as marriage, study abroad, employment, and medical tourism has been on the rise recently. In 2020, the number slowed for a while due to the COVID-19 pandemic, but it continues to rise again, making it appropriate to call Korea a multicultural society [1]. Entering into a multicultural society clearly shows that the use of medical institutions by foreigners residing in our society is also increasing. In this context, nurses need skills to communicate therapeutically with foreigners from various cultures [2, 3]. Furthermore, cultural capability related to accepting multiculturalism, respecting and empathizing with other cultures, and cultivating multicultural attitudes is becoming an indispensable area of competency for nurses [4, 5].

The awareness of the utilization rate of medical institutions in Korea was investigated targeting foreigners who came from various cultures. It was found that while they had positive thoughts related to the level of medical care and accessibility, they felt that medical professionals in Korea lack understanding of cultural empathy or multicultural attitudes [2]. Compared to Koreans, who are traditionally homogeneous, foreigners from various cultures have very different cultural demands and belief systems. Accordingly, it is necessary to provide nursing services suitable for their cultural characteristics [6, 7]. In order to provide nursing services to foreign patients, cultural empathy should be cultivated, and nurses should be able to provide holistic care that considers the cultural values, beliefs, traditions, customs, and lifestyles of foreign patients [8, 9]. To this end, acceptance of multiculturalism can be seen as very important. Multicultural acceptance is a basic ability required to provide effective nursing care to foreign patients with diverse cultural backgrounds, referring to the degree to which multicultural awareness is accepted from multiple angles [2].

Recently, in response to the growing trend of multicultural acceptance of health care services, some nursing colleges are implementing contents related to multicultural nursing in their regular curriculum [10, 11]. Despite offering a regular curriculum based on the importance of cultural empathy and attitude toward multicultural foreign patients, it was reported that nursing students' multicultural acceptance, cultural empathy, and multicultural attitude remain low [12]. Furthermore, in the case of new nurses working in clinical practice after graduation, excessive work burden leads to a lack of understanding of multicultural acceptance, and it is becoming a big task to perform efficient nursing considering the cultural differences among foreign patients.

Nevertheless, previous studies on multicultural acceptance among Korean nurses and nursing students including those of Peek and Park [3], Cho and Sok [4], and Hwang, Chun, and Hur [10] only evaluated cultural competence through multicultural awareness. In addition, studies investigating nursing students' multicultural acceptance, cultural empathy, and multicultural attitudes in nursing practice remain deficient [2–4].

In our changing society, acceptance of multiculturalism can be considered an essential qualification and capability that nurses should further have [11]. In improving therapeutic communication skills with multicultural foreign patients, it is very important to accept desirable multiculturalism and develop cultural empathy and multicultural attitudes [4, 5, 11]. Above all, it is most urgent to systematically include multicultural nursing education in the regular curriculum for nursing students to develop critical competencies in advance to perform high-quality nursing for foreign patients.

This study presents detailed plans for an effective multicultural nursing education program for nursing students who will become health managers in the future. At the same time, this study ultimately contributes to fostering competent nurses with expertise in multicultural nursing.

The purpose of this study was to examine and identify the factors influencing the degree of multicultural acceptance of Korean nursing students. The aims of the study were: (1) to identify the general characteristics of Korean nursing students; (2) to examine the degree of multicultural acceptance and factors related to it; (3) to examine the differences of the degrees of multicultural acceptance according to the general characteristics of Korean nursing students; (4) to examine the correlations between multicultural acceptance and factors related to it; (5) to determine the factors that influence the degree of multicultural acceptance among Korean nursing students.

## Methods

### Design, setting and participants

A cross-sectional descriptive design was employed. Study participants included 147 nursing students at two Universities in Seoul or Gyeonggi-do, South Korea. Study participants were recruited using a convenience sampling method regardless of grade. Researchers did not recruit study participants with special emphasis on specific grades or classes. The eligibility criteria were: consented to participate in this study, understood the purpose of this study, had the capability to understand Korean verbally. Of the 152 questionnaires, 152 were answered. Due to incomplete data, only a total of 147 (96.71%) questionnaire responses were included in the final dataset. Sample size adequacy (*n* = 138) using G power 3 analysis software was estimated based on an alpha level = 0.05, medium effect size = 0.15, and power = 0.95 [13]. The sample size of the study was adequate.

### Instruments

General characteristics of study participants based on a literature review and previous research [2–4, 10–12] included gender, age, religion, economic status, living in the last 5 years, living together, foreign friends, Foreign residence experience, multicultural education, multicultural internet and media accessibility, and foreign language skills. They were binary or categorical variables as a total of 11 items. Of them, economic status was categorized in 3 levels, and the 3 categories were selected and determined by subjectivity of study participants.

The multicultural acceptance scale developed by Kim [14] was used in this study. It consists of a total of 45 questions, including acceptance of multiculturalism, sensitivity, and openness to multiculturalism. As a 5-point Likert scale, the score ranges from a minimum of 45 to a maximum of 225, and a higher score means a higher level of multicultural acceptance of the study participants. At the time of the development [14], Cronbach’s α = 0.84, and reliability in this study was Cronbach’s α = 0.94.

The cultural empathy scale that Kim [15] used and modified to suit the Korean situation was finally adapted and underwent the validation process. This scale consists of a total of 20 questions, including 9 emotional empathy items and 11 cognitive empathy items. As a 5-point Likert scale, the score ranges from a minimum of 20 to a maximum of 100, and a higher score means a higher level of cultural empathy of the study participants. At the time of the development [15], Cronbach’s α = 0.85, and reliability in this study was Cronbach’s α = 0.93.

The multicultural attitude scale used was developed and verified through exploratory factor analysis by Seon [16]. It consists of a total of 29 questions, including 10 questions on multicultural openness, 10 on multicultural acceptance, and 9 on multicultural coexistence. As a 6-point Likert scale, the score ranges from a minimum of 29 to a maximum of 174, and a higher score means a higher level of multicultural attitude of the study participants. At the time of the development [16], Cronbach’s α = 0.85, and reliability in this study was Cronbach’s α = 0.93.

Multicultural acceptance, cultural empathy, and multicultural attitude were continuous variables.

### Data collection

This study was conducted from March to June 2021. The directors of nursing schools at two Universities were contacted to request for cooperation and conduct the study. Study participants were provided with a detailed information and a consent form for this study. A researcher directly distributed the self-report questionnaires and collected the completed questionnaires. When collecting data, social distancing was observed to ensure the safety of data collection due to COVID-19, and participants were asked to wear masks properly and fill out questionnaires. The questionnaire was self-reported and took about 20–25 min to complete.

### Ethical considerations

This study was approved by the Institutional Review Board of D University (IRB No. 1040656–202005-SB-01–01). After the approval of Institutional Review Board, the purpose and procedure of this study were explained to study participants, and it was revealed that it would not be used for any purpose other than research purposes. The provision of personal information and confidentiality related to anonymity were explained. It was explained that there was no disadvantage in not participating in the study or withdrawing from participation in the middle. In addition, it was explained that they could withdraw at any time during participation, and written consent forms were obtained for data collection. A questionnaire was distributed to nursing students who wished to voluntarily participate in the study.

### Data analysis

SPSS PC + version 23.0 statistical software program analyzed the data from this study. For the reliability of the measurement tool, the reliability coefficient was obtained using Cronbach's-α, an internal consistency test. The descriptive statistics using frequency, percentage, mean, standard deviation analyzed the general characteristics of the study participants and levels of study variables. The t-test, ANOVA, and Scheffe post hoc test analyzed the differences on multicultural acceptance, cultural empathy, and multicultural attitude according to the general characteristics of the study participants. Pearson’s correlation coefficient analyzed the correlations between multicultural acceptance and related factors. Hierarchial stepwise multiple regression statistics analyzed and examined the factors influencing the degree of multicultural acceptance. In Stage 1, the general characteristics of the study participants were input into hierarchical stepwise multiple regression statistics as independent variables. In Stage 2, cultural empathy and multicultural attitude were analyzed by entering the hierarchical stepwise multiple regression statistics as independent variables along with the general characteristics of the study participants. Statistically significant level of a *p*-value was the less than 0.05.

## Results

### Demographic and other data of study participants

Table 1 shows demographic and other data of study participants in this study.

**Table 1:** Demographic and other data of study participants

Characteristics	Frequency	Percentage
Gender		
Male	32	21.8
Female	115	78.2
Age (year)		
< 22	106	72.1
22∼24	15	10.2
≥ 25	26	17.7
Religion		
Yes	26	17.7
No	121	82.3
Economic status		
Good	7	4.8
Moderate	128	87.1
Bad	12	8.1
Living in the last 5 years		
City	139	94.6
Country	8	5.4
Living together		
With family	143	97.3
Others (Dormitory)	4	2.7
Foreign friends		
Yes	48	32.7
No	99	67.3
Foreign residence experience		
Yes	26	17.7
No	121	82.3
Multicultural education experience		
Yes	123	83.7
No	24	16.3
Multicultural internet and media accessibility		
High	45	30.6
Moderate	92	62.6
Low	10	6.8
Foreign language skills		
Good	9	6.1
Moderate	101	68.7
Bad	37	25.2

### Levels of multicultural acceptance, cultural empathy, and multicultural attitude

In this study, the multicultural acceptance of study participants was 176.43 points based on a median of 174.00 points, and cultural empathy was 79.68 points based on a median of 77.00 points. Multicultural attitude was 139.92 points based on the median 138.00 points. Mean scores for multicultural acceptance, cultural empathy, and multicultural attitude were a slightly higher levels when compared to the median values of the score ranges (Table 2).

**Table 2:** Levels of cultural empathy, multicultural attitude, and multicultural acceptance

Variables	Range (Median value)	Min	Max	Mean (SD)
Multicultural acceptance	45–225 (174)	134.00	212.00	176.43 (18.33)
Cultural empathy	20–100 (77)	60.00	100.00	79.68 (11.04)
Multicultural attitude	29–174 (138)	105.00	174.00	139.92 (17.66)

### Differences on multicultural acceptance, cultural empathy, and multicultural attitude according to the general characteristics of study participants

Regarding multicultural acceptance, there were statistically significant differences in the living together (t = -0.89, *p* = 0.017), multicultural internet and media accessibility (F = 20.63, *p* < 0.001), and foreign language skills (F = 3.67, *p* = 0.028). The multicultural acceptance was higher in the nursing students living with family than them living in dormitory. In terms of multicultural internet and media accessibility, multicultural acceptance was higher in the high accessibility group than in the moderate or low accessibility group. As for Foreign language skills, the multicultural acceptance was higher in the good foreign language skills group than in moderate or bad foreign language skills group.

In terms of cultural empathy, religion (t = -4.25, *p* = 0.024), economic status (F = 6.23, *p* = 0.003), foreign friend (t = 1.47, *p* = 0.016), multicultural education experience (t = 4.50, *p* < 0.001), and multicultural internet and media accessibility (F = 17.03, *p* < 0.001) showed statistically significant differences. In terms of religion, the no-religious group had higher cultural empathy than the religious group. In terms of economic status, the bad economic status group had higher cultural empathy than the good economic status group. In terms of foreign friend, the foreign friend group had higher cultural empathy than the non- foreign friend group. In terms of multicultural education experience, multicultural education experience group had higher cultural empathy than the no experience group. In terms of multicultural internet and media accessibility, the high accessibility group of multicultural internet and media had higher cultural empathy than the moderate or low group.

In terms of multicultural attitude, age (F = 6.26, *p* = 0.002), economic status (F = 3.66, *p* = 0.028), living together (t = -0.07, *p* = 0.008), and multicultural internet and media accessibility (F = 14.43, *p* < 0.001) showed statistically significant differences. In terms of age, the 22–24 years old group had higher multicultural attitude than the 25 years old or above group. In terms of economic status, the bad economic status group had higher multicultural attitude than the good economic status group. In terms of living together, the multicultural attitude was higher in the nursing students living with family than them living in dormitory. In terms of multicultural internet and media accessibility, the high accessibility group of multicultural internet and media had higher multicultural attitude than the moderate or low group (Table 3).

**Table 3:** Differences of multicultural acceptance, cultural empathy, and multicultural attitude according to the general characteristics of the study participants

Variables	Multicultural acceptance		Cultural empathy		Multicultural attitude	
	Mean (SD)	t or F (P) Scheffe	Mean (SD)	t or F (P) Scheffe	Mean (SD)	t or F (P) Scheffe
Gender						
Male	166.19 (19.60)	-2.95 (0.286)	72.13 (10.72)	-3.32 (0.616)	132.69 (17.46)	-1.17 (0.775)
Female	176.72 (17.36)		79.22 (10.66)		136.82 (17.68)	
Age (year)						
< 22^a^	175.42 (19.78)	1.04 (0.357)	78.46 (11.48)	1.33 (0.269)	137.31 (17.42)	6.26 (0.002*) b > c
22∼24^b^	175.60 (7.24)		77.53 (5.63)		143.40 (9.67)	
≥ 25^c^	169.73 (16.17)		74.54 (11.30)		125.92 (18.68)	
Religion						
Yes	171.38 (17.11)	-0.93 (0.194)	69.77 (7.17)	-4.25 (0.024*)	128.69 (15.75)	-2.34 (0.210)
No	175.08 (18.58)		79.37 (11.00)		137.47 (17.72)	
Economic status						
Good^a^	169.71 (6.34)	0.34 (0.713)	67.29 (3.90)	6.23 (0.003*) a < c	120.57 (21.47)	3.66 (0.028*) a < c
Moderate^b^	174.45 (18.70)		77.55 (10.82)		136.13 (17.27)	
Bad^c^	176.92 (19.44)		85.08 (11.37)		142.67 (15.58)	
Living in the last 5 years						
City	173.50 (18.01)	-2.60 (0.757)	77.66 (10.97)	-0.05 (0.327)	135.49 (17.40)	-1.23 (0.131)
Country	190.50 (17.30)		77.86 (12.99)		143.38 (21.72)	
Living together						
With family	182.50 (31.75)	-0.89 (0.017*)	77.40 (11.01)	-1.82 (0.456)	136.50 (31.75)	-0.07 (0.008*)
Others (Dormitory)	174.20 (17.95)		87.50 (7.51)		135.90 (17.30)	
Foreign friend						
Yes	180.85 (15.13)	3.04 (0.078)	79.58 (9.40)	1.47 (0.016*)	139.77 (16.52)	1.86 (0.274)
No	171.31 (18.99)		76.75 (11.68)		134.05 (17.97)	
Foreign residence experience						
Yes	178.81 (12.95)	1.35 (0.066)	77.69 (12.14)	0.01 (0.133)	138.77 (17.49)	0.91 (0.722)
No	173.49 (19.20)		77.67 (10.84)		135.31 (17.71)	
Multicultural education experience						
Yes	176.64 (18.03)	3.44 (0.312)		4.50 (< 0.001*)	137.84 (17.51)	3.07 (0.597)
No	163.08 (15.74)		68.96 (3.59)		126.08 (15.26)	
Multicultural internet and media accessibility						
High^a^	186.96 (15.96)	20.63 (< 0.001*) a > c	84.47 (11.61)	17.03 (< 0.001*) a > b,c	147.04 (15.72)	14.43 (< 0.001*) a > b,c
Moderate^b^	169.79 (15.68)		75.34 (9.39)		131.10 (16.48)	
Low^c^	160.70 (22.47)		68.60 (6.93)		130.20 (14.54)	
Foreign language skills						
Good^a^	188.00 (9.80)	3.67 (0.028*) a > c	83.33 (9.89)	1.82 (0.165)	143.22 (7.64)	2.16 (0.119)
Moderate^b^	174.82 (19.23)		76.71 (11.90)		136.91 (19.41)	
Bad^c^	170.05 (15.74)		78.92 (8.19)		131.43 (12.99)	

^*^p < 0.05

^a,b,c^Items included for Scheffe post hoc test

### Correlations between multicultural acceptance and factors related it

Multicultural acceptance showed statistically significant positive correlations with cultural empathy (γ = -0.66, *p* < 0.01) and multicultural attitude (γ = -0.65, *p* < 0.01). It can be seen that the higher the study participant's multicultural acceptance, the higher the cultural empathy and multicultural attitude (Table 4).

**Table 4:** Correlations between multicultural acceptance and the study variables

Variables	Multicultural acceptance	Cultural empathy	Multicultural attitude
	γ (*p*)		
Multicultural acceptance	1		
Cultural empathy	0.66 (< 0.01*)	1	
Multicultural attitude	0.65 (< 0.01*)	0.66 (< 0.01*)	1

^*^p < 0.05

### Factors influencing multicultural acceptance

The assumptions of the regression model were verified to determine whether this study is suitable for regression analysis. To verify the independence of the residuals with Durbin-Watson, the autocorrelation of the error of the statistic was tested, which satisfied the assumption of the regression equation at 1.73. In addition, the tolerance limit was 0.26 to 0.78, which was more than 0.10. The variance inflation factor (VIF) was 1.33 to 3.84, which was not greater than 10. There was no problem with multicollinearity for all variables.

Factors influencing multicultural acceptance of nursing students were analyzed by hierarchical regression analysis. First, general characteristics were input into the one stage regression model in terms of factors affecting multicultural acceptance of nursing students, which were statistically significant (F = 7.07,* p* < 0.001). Variables that appeared statistically significant were the residential area where they lived for the past 5 years (β = 0.23, *p* = 0.006), whether they have foreign friends (β = -0.27, *p* = 0.001), experience staying abroad (β = -0.26, *p* = 0.003), multicultural education experience (β = -0.18, *p* = 0.025), and access to the Internet and media for multiculturalism (β = -0.30, *p* < 0.001). The explanation power of the one-stage regression model was 33.3%. In the two-stage regression model, general characteristics and major variables such as cultural empathy and multicultural attitude were input (F = 28.48, *p* < 0.001). Statistically significant variables were the residential area where they lived for the past 5 years (β = 0.19, *p* = 0.001), whether they have foreign friends (β = 0.16, *p* = 0.003), multicultural education experience (β = 0.02, *p* = 0.002), access to the Internet and media for multiculturalism (β = 0.17, *p* = 0.003), cultural empathy (β = 0.55, *p* < 0.001), and multicultural attitude (β = 0.26, *p* = 0.001). The explanation power of the two-stage regression model increased by 39.2% compared to the one-stage model.

The important variables influencing multicultural acceptance of nursing students were cultural empathy, followed by multicultural attitude, residential area where they lived for the past 5 years, accessibility to multicultural internet and media, whether they have foreign friends, and multicultural education experience. The explanation power of the final regression model was 72.5% (Table 5).

**Table 5:** Factors influencing multicultural acceptance

Variables	Stage 1					Stage 2				
	B	SE	β	*t*	*p*	B	SE	β	*t*	*p*
Gender	8.36	3.45	0.19	2.13	0.067	4.03	2.26	0.09	1.78	0.077
Age (year)	-1.68	2.21	-0.07	-0.76	0.449	1.09	1.43	0.05	0.76	0.450
Religion	6.31	4.05	0.13	1.56	0.121	-1.84	2.66	-0.04	-0.69	0.490
Economic status	-2.13	4.41	-0.04	-0.48	0.631	-2.43	1.71	-0.08	-1.42	0.157
Living in the last 5 years	18.56	6.58	0.23	2.82	0.006*	15.31	4.32	0.19	3.54	0.001*
Living together	-4.68	8.58	-0.04	-0.55	0.586	-8.87	5.62	-0.08	-1.58	0.117
Foreign friends	-10.37	3.20	-0.27	-3.25	0.001*	6.33	2.13	0.16	2.98	0.003*
Foreign residence experience	-12.36	4.09	-0.26	-3.02	0.003*	-2.85	2.72	-0.06	-1.05	0.297
Multicultural education experience	-8.91	3.94	-0.18	-2.26	0.025*	1.19	2.63	0.02	1.45	0.002*
Multicultural internet and media accessibility	-9.86	2.51	-0.30	-3.93	< 0.001*	8.65	2.89	0.17	2.99	0.003*
Foreign language skills	0.11	2.75	0.01	0.04	0.968	-1.65	1.83	-0.05	-0.90	0.369
Cultural empathy						0.92	0.14	0.55	6.50	< 0.001*
Multicultural attitude						0.27	0.08	0.26	3.36	0.001*
Adj R^2^ = 0.333, F = 7.07, *p* < 0.001*						Adj R^2^ = 0.725, F = 28.48, *p* < 0.001*				

Stage 1,2 = order of input into hierarchical stepwise multiple regression statistics

Input variables in stage 1 = gender, age, religion, economic status, living in the last 5 years, living together, foreign friends, foreign residence experience, multicultural education experience, multicultural internet and media accessibility, foreign language skills

Input variables in stage 2 = gender, age, religion, economic status, living in the last 5 years, living together, foreign friends, foreign residence experience, multicultural education experience, multicultural internet and media accessibility, foreign language skills, cultural empathy, multiple attitude

^*^p < 0.05

## Discussions

In this study, the degree of multicultural acceptance, cultural empathy, and multicultural attitude was relatively higher than the median value, which was similar to the findings of Cho, Sok [4], Jeon, Hwang [17], and Rho, Jung [18]. With the rapid development of mass media, exchanges with other cultures are actively taking place through the media, Internet, and SNS. The need to take care of foreign patients and their families from various cultures has likewise increased in the domestic nursing field. Consequently, it is considered that interest in multicultural nursing has increased more than ever [19, 20].

Next, multicultural acceptance and multicultural attitudes differ significantly according to Internet and media accessibility, and foreign language skills. In the case of cultural empathy, there is a significant difference depending on whether nursing students have foreign friends and multicultural education experiences. This is similar to the research results of Cho, Sok [4] and Kim [21] and Jeon, Ko [22], and indicates that if access to the Internet and media for multiculturalism is high, one can have a deeply sympathetic attitude by recognizing the rapidly changing reality and developing an active interest in various cultural societies and positive openness, even if there is no special interest in multiculturalism [23–25]. In this regard, language barriers with foreign patients can hinder the provision of nursing services since communication with patients is an essential element [26, 27]. Therefore, detailed measures are needed to improve the foreign language ability anchored on practical nursing for nursing students to increase multicultural nursing competency.

Through this study, it can be seen that multicultural acceptance, cultural empathy, and multicultural attitude are positively correlated, also validating the research results of Kim [21], Noh, and Lee [28]. This suggests that increasing the breadth of understanding of various cultural capabilities is required to establish acceptance and attitude toward multiculturalism. It is necessary to broaden the understanding of the physical, psychological, and social health of foreign patients from various cultures in advance by expanding the nursing students’ awareness of cultural differences [29–31].

Finally, the important variables influencing multicultural acceptance of nursing students as shown in this study were cultural empathy, followed by multicultural attitude, residential area where they lived for the past 5 years, access to the Internet and media for multiculturalism, and multicultural education experience. Overall, a systematic multicultural nursing education program in the regular curriculum for nursing students should be developed to increase acceptance of multiculturalism. Toward this end, measures to raise awareness of multiculturalism and cultural competency of nursing students should be considered together [29, 30]. In particular, it is urgent to develop nursing education contents that enable nursing students to understand and accommodate multicultural awareness more comprehensively [30, 32, 33].

Cultural empathy should be enhanced and multicultural attitudes should be positively changed to improve multicultural acceptance of nursing students. Strategies to strengthen volunteer activities or experiential learning programs for foreigners from various cultures should also be considered. In addition, a broader understanding and specific design of effective multicultural education programs are required to further expand access to the Internet and media for multiculturalism. It is necessary to develop professional and skilled multicultural nursing education content programs in the nursing education curriculum, and to systematically operate and utilize them. Since there are very few previous studies related to multicultural acceptance of nursing students, repeated studies with an expanded number of subjects should be conducted in the future. Furthermore, based on the results of this study, an experimental study to develop and apply a multicultural nursing curriculum program for nursing students is needed.

## Limitations

Previous studies related to multicultural aspects in relation to nursing students are insufficient. Care should be taken in expanding the results of this study to explain the factors that affect multicultural acceptance of all nursing students in Korea. Also, study limitations can be expanded to include the cross-sectional nature of the study, study participants were not "blinded" from the purpose of the study, so there may have been some bias introduced. And a small sample size from one or two region/institution and convenience sampling can be study limitations.

## Conclusion

In conclusion, the variables influencing multicultural acceptance of nursing students in this study were cultural empathy, multicultural attitude, the residential area where they lived for the past 5 years, access to the Internet and media for multiculturalism, whether they have foreign friends, and multicultural education experience. The most important variable affecting multicultural acceptance of nursing students was cultural empathy. This study is meaningful in that the findings can be used as basic data for the development of a multicultural nursing education curriculum for nursing students.

## Data Availability

The datasets generated and/or analyzed during the current study are not publicly available due no new data were created or analyzed in this study, but are available from the corresponding author on reasonable request.
