# Correction: factors influencing multicultural acceptance of Korean nursing students

**DOI:** 10.1186/s12912-023-01652-8

**Published:** 2024-01-02

**Authors:** Minkyung Gu, Sohyune Sok

**Affiliations:** 1 Department of Nursing, College of Health Science, Daejin University, Pocheon-Si, Gyeonggi-Do Republic of Korea; 2 College of Nursing Science, Kyung Hee University, 26, Kyungheedae-Ro, Dongdaemun-Gu, 02447 Seoul, Republic of Korea

**Correction to: BMC Nursing (2023) 22:427**

10.1186/s12912-023-01583-4

Following publication of the original article [1], it was reported that reference number six needed to be updated.

The original version was:

Gisela T, Pedro L, Filomena G. International portuguese nurse leaders’ insights for multicultural nursing. Int J Environ Res Public Health. 2022;19(19):12,144. 10.3390/ijerph191912144.

The correct version is:

Teixeira G, Lucas P, Gaspar F. International portuguese nurse leaders’ insights for multicultural nursing. Int J Environ Res Public Health. 2022;19(19):12,144. 10.3390/ijerph191912144.

The original article [1] has been corrected.
